# Gut microbiome combined with metabolomics reveals biomarkers and pathways in central precocious puberty

**DOI:** 10.1186/s12967-023-04169-5

**Published:** 2023-05-11

**Authors:** Xiaoyan Huang, Jixiong Chen, Haozhe Zou, Peng Huang, Hailing Luo, Haidan Li, Yuhua Cai, Li Liu, Yongsheng Li, Xiaojie He, Wei Xiang

**Affiliations:** 1 Department of Genetics, Metabolism and Endocrinology, Hainan Women and Children’s Medical Center, Haikou, Hainan China; 2 Department of Medical Care Center, Hainan Provincial People’s Hospital, Haikou, Hainan China; 3 College of Biomedical Information and Engineering, Hainan Medical University, Haikou, Hainan China; 4 Department of Pediatrics, The Second Xiangya Hospital, Central South University, Changsha, China; 5 Children’s Brain Development and Brain Injury Research Office, The Second Xiangya Hospital, Central South University, Changsha, China; 6 Institute of Pediatrics, The Second Xiangya Hospital, Central South University, Changsha, China

**Keywords:** Central precocious puberty, Gut microbes, Metabolites, Multi-omics data, Machine-learning

## Abstract

**Background:**

Central precocious puberty (CPP) is a common disease in prepubertal children and results mainly from disorders in the endocrine system. Emerging evidence has highlighted the involvement of gut microbes in hormone secretion, but their roles and downstream metabolic pathways in CPP remain unknown.

**Methods:**

To explore the gut microbes and metabolism alterations in CPP, we performed the 16S rRNA sequencing and untargeted metabolomics profiling for 91 CPP patients and 59 healthy controls. Bioinformatics and statistical analyses, including the comparisons of alpha and beta diversity, abundances of microbes, were undertaken on the 16S rRNA gene sequences and metabolism profiling. Classifiers were constructed based on the microorganisms and metabolites. Functional and pathway enrichment analyses were performed for identification of the altered microorganisms and metabolites in CPP.

**Results:**

We integrated a multi-omics approach to investigate the alterations and functional characteristics of gut microbes and metabolites in CPP patients. The fecal microbiome profiles and fecal and blood metabolite profiles for 91 CPP patients and 59 healthy controls were generated and compared. We identified the altered microorganisms and metabolites during the development of CPP and constructed a machine learning-based classifier for distinguishing CPP. The Area Under Curves (AUCs) of the classifies were ranged from 0.832 to 1.00. In addition, functional analysis of the gut microbiota revealed that the nitric oxide synthesis was closely associated with the progression of CPP. Finally, we investigated the metabolic potential of gut microbes and discovered the genus Streptococcus could be a candidate molecular marker for CPP treatment.

**Conclusions:**

Overall, we utilized multi-omics data from microorganisms and metabolites to build a classifier for discriminating CPP patients from the common populations and recognized potential therapeutic molecular markers for CPP through comprehensive analyses.

**Supplementary Information:**

The online version contains supplementary material available at 10.1186/s12967-023-04169-5.

## Background

The sign of the initiation of puberty is the reactivation of the hypothalamic-pituitary–gonadal axis (HPGA) [1]. Central precocious puberty (CPP) is due to the early release of gonadotropin-releasing hormone (GnRH), which causes HPGA to activate prematurely. Precocious puberty could accelerated bone development, result in premature discontinuation of linear growth [2], and increase the risk of type 2 diabetes [3, 4], obesity, cardiovascular disease [5], and breast cancer [6, 7]. The prevalence of CPP is 5 to 10 times higher prevalence in girls than in boys [8]. Globally, at least 0.2% of women experienced earlier puberty each year [9]. However, the pathogenesis of CPP is not completely known and remains to be studied.

The microbiota-gut-brain axis (MBGA) refers to that gut microbiota affects the central nervous system by regulating intestinal neural, endocrine, and immunologic pathways. Moreover, this manner is often bidirectional [10]. The role of gut microbiota on the host is not limited to modulating the host immunity, nervous and hormones [11], but also regulating intestinal epithelial cells the blood brain barrier [12], and the production and degradation of neuroactive compounds [13], such as Nitrogen Monoxide (NO) [14]. Microbial metabolites involved in MBGA include γ-aminobutyric acid (GABA), serotonin, butanoate, cortisol, and quinolinic acid [15]. With the inclusion of extensive studies, the mechanism of interaction between gut microbiota and brain is becoming more and more clear. It’s worth noting that even though the relationship between precocious puberty and gut microbiota has been investigated [16], the complex association between gut microbiota, metabolites, and CPP is largely unknown.

In this study, we dissected gut microbiome and metabolomics profiles from 150 participants to explore the correlations between gut microbiome features, metabolic features, and CPP. Bioinformatics and statistical analyses, including the comparisons of alpha and beta diversity, abundances of microbes, were undertaken on the 16S rRNA gene sequences and metabolism profiling. We revealed the widespread alterations of gut microbes and metabolites in CPP, which were involved in nitric oxide synthesis pathway. The results provided novel insights into recognizing potential therapeutic molecular markers for CPP.

## Methods

### Participants

In total, 150 fecal and blood samples (91 CPP patients, 59 healthy controls) were recruited from the Hainan women and Children’s medical center, Hainan Medical University. Patients who satisfied the following criteria were enrolled in the CPP group: (1) Girls younger than 10 years old. (2) Complying with the diagnostic criteria in the diagnosis and treatment guidelines of CPP: a) Secondary sexual characteristics appeared before 8 years in girls and progressed according to the normal developmental routine. (b) With evidence of gonadal development. (c) The height growth spurt during the development. (d) The gonadotropin elevated to pubertal level and luteinizing hormone-releasing hormone (LHRH) provocation test was positive. (e) The bone age was advanced 1 year more than the chronological age.

Participants were excluded when they met any of the following criteria. (a) Patients with other systemic diseases, including underlying diseases with clinical impacts (such as serious diseases of the heart, liver, kidney, lungs, and brain), tumors, abnormal glucose metabolism, immunodeficiency, and suffering tuberculosis, hepatitis B and C and other diseases within half a year. (b) Any history of other drugs, such as various antibiotics, used other than immune-pharmaceuticals (for instance, prednisone, tacrolimus cyclosporin, and cyclophosphamide) within 3 months. (c) Patients undergoing major gastrointestinal, inflammatory bowel diseases, long-term constipation, or diarrhea. (d) Coexisting other connective tissue diseases (such as Sjogren’s syndrome and overlap syndrome).

### Sample collection and preparation

The subjects’ feces (greater than 400 mg) were collected into a sterile preservation tube with a sterile spoon, the Bristol Stool Scale scores were recorded. Then the feces were immediately placed into a – 80 °C freezer for cryopreservation for testing gut microbial and metabolite. About 3 ml of whole blood samples of the same subjects were collected using heparin anti-coagulant tubes. After staying still for 30 min at room temperature, all samples were centrifuged at 1300–2000 g for 10 min at 4 °C. After removing the upper plasma (not less than 0.3 ml), the samples were flash-frozen in liquid nitrogen followed by and preserved at − 80 °C to detect blood metabolites.

### DNA library construction and sequencing

After extracting genomic DNA from the fecal samples using CTAB or SDS methods, the V4 variable region of 16S rDNA was amplified by PCR utilizing primers specific for Barcode and high-fidelity DNA polymerase. The library was quantified by Qubit and Q-PCR after construction by using TruSeq^®^ DNA PCR-Free Sample Preparation Kit. Sequencing of the V4 variable region was performed through Illumina Miseq after the library was qualified.

### 16S rRNA gene sequencing data analysis

All the raw 16 s rRNA sequencing data were processed using QIIME software [17]. The sequences with 97% resemblance for each sample were clustered into operational taxonomic units (OTUs) through Usearch algorithm [18]. Then based on the reference sequence of the Silva database [19], the OTUs representative sequence was used for species annotation using the UCLUST algorithm [18]. The Chao index measured in OTU was used to evaluate alpha diversity. Beta diversity was calculated through the Bray–Curtis and was used to build principal coordinate analysis (PCoA). ANOSIM test was carried out to visualize and compare the differences in beta diversity between CPP and healthy control groups.

### Untargeted metabolomics profiling

To identify the metabolomic features of subjects' fecal and blood samples, untargeted metabolomic analysis methods were performed using an ultra-performance liquid chromatography system with quadrupole-time-of-flight mass spectrometry (UPLC-QTOFMS), which was used to measure polar metabolites, such as organic acids.

### LC–MS/MS analysis

All samples were separated by Ultra-high-performance liquid chromatography (UHPLC) reversed separation on the Agilent 1290 Infinity UHPLC. The conditions for detection were as follows: the temperature was 25 °C, the flow rate was 0.5 mL/min, and the sample injection volume was 2 μL. A mobile phase consisting of a binary solution was used: Mobile phase A contained water, 25 mM ammonium acetate and 25 mM ammonia, and Mobile phase B consisted of acetonitrile. The gradient elution process was as below: 95% B for 0–0.5 min, the concentration of B from 95 to 65% linearly in 0.5–7 min, B from 65 to 40% linearly for 7–8 min, B was kept at 40% for 8–9 min; B was changed from 40 to 95% linearly for 9–9.1 min, lastly, B remained at 95% for 9.1–12 min. The samples were placed within an autosampler at 4 °C throughout the analysis. To avoid the influences due to the signal fluctuations arising from the detection of the instrument, the samples were analyzed continuously in random order. QC samples were inserted into the sample cohort to monitor and assess the solidity of the system and the credibility of experimental data.

### Quadrupole–time-of-flight conditions

Positive and negative ions were separated through hydrophilic interaction chromatography (HILIC), followed by UHPLC separation, then a Triple TOF^®^ 6600 (AB SCIEX) was intended for the mass spectrometer. The ESI operating conditions were as below: nebulization pressure (Gas1) was set as 60, adjuvant air pressure (Gas2) was 60, curtain gas was 30, ion source temperature was 600 ℃, the ion spray voltage was 5500 V in the positive ion mode and − 5500 V in the negative ion mode, the m/z range of TOF MS and daughter ion scanning were 0.20 s/spectra and 0.05 s/spectra, respectively. Secondary mass spectrometry was acquired by information-dependent acquisition (IDA) with the high sensitivity mode: the declustering potential (DP) was ± 60 V (positive and negative mode), the collision energy was 35 ± 15 eV, excluding isotopes within 4 Da, and the candidate ions to monitor per cycle: 10.

### Random forest classification

To identify biomarkers in gut microorganisms, fecal metabolites, and blood metabolites that could be used to distinguish CPP patients from the population, a random forest model based on gut microorganisms, fecal metabolites, and blood metabolites was constructed using the *R* package “randomForest” (Version 4.7–1.1) to identify the important features. The combined dataset of the CPP group and healthy control group was randomly split into the training set and testing set with a ratio of 7:3. In addition, the Boruta algorithm of the R package “Boruta” (Version 7.0.0) was used to select gut microorganisms, fecal metabolites, and blood metabolites that could make significant contributions to the classification, and based on the selected features constructed a model. The area under curve (AUC) of the receiver operating characteristic (ROC) curve was plotted using the R package “pROC” (Version 1.18.0) to evaluate the model performance.

### Metagenomic metabolomic pathway prediction by PICRUSt2

The pathways of the gut microbiome and the activity of gut-brain modules were predicted by PICRUSt2 [20]. Differences in pathways abundances between the CPP and healthy control groups were calculated by* t*-test, and *p*-values were corrected applying Benjamini-Hochberg (BH) adjustment.

### Metabolomic pathway enrichment

According to the Kyoto Encyclopedia of Genes and Genomes (KEGG) metabolite database [21], we used hypergeometric tests to perform the functional annotation of fecal and blood metabolites, and Benjamini-Hochberg (BH) adjustment was applied to correct *p*-values. The pathways were considered as significantly enriched only if the number of their corresponding differential metabolites was at least 3 and their corrected *p*-values were less than 0.05.

### 16S and metabolome correlation analysis

We used correlation analysis to identify differential metabolites associated with differential microorganisms. The Spearman correlation coefficient was calculated. Only the differential metabolites with the correlation coefficients *R*^2^ ≥ 0.3 or* R*^2^ ≤ − 0.3, and BH corrected *p-*values < 0.05 were selected as metabolites potentially affected by microorganisms.

### Statistical analysis

All statistical analysis and charting were performed using R software (version 4.1.2). Kolmogorov–Smirnov test was used to check the normal distribution of the data. Wilcoxon’s rank-sum test was used to calculate the differences in metabolite and microbial abundances, which are non-normally distributed. The Spearman correlation coefficient was determined by the *R* package “corrplot”, with a corrected significance threshold of *p* < 0.05. Additionally, partial least square discriminant analysis (PLS-DA) was used to analyze the between-group difference. BH-adjusted *p*-values < 0.05 was considered statistically significant.

## Results

### Gut microbiota helps to effectively distinguish CPP patients from controls

**Fig. 1:**
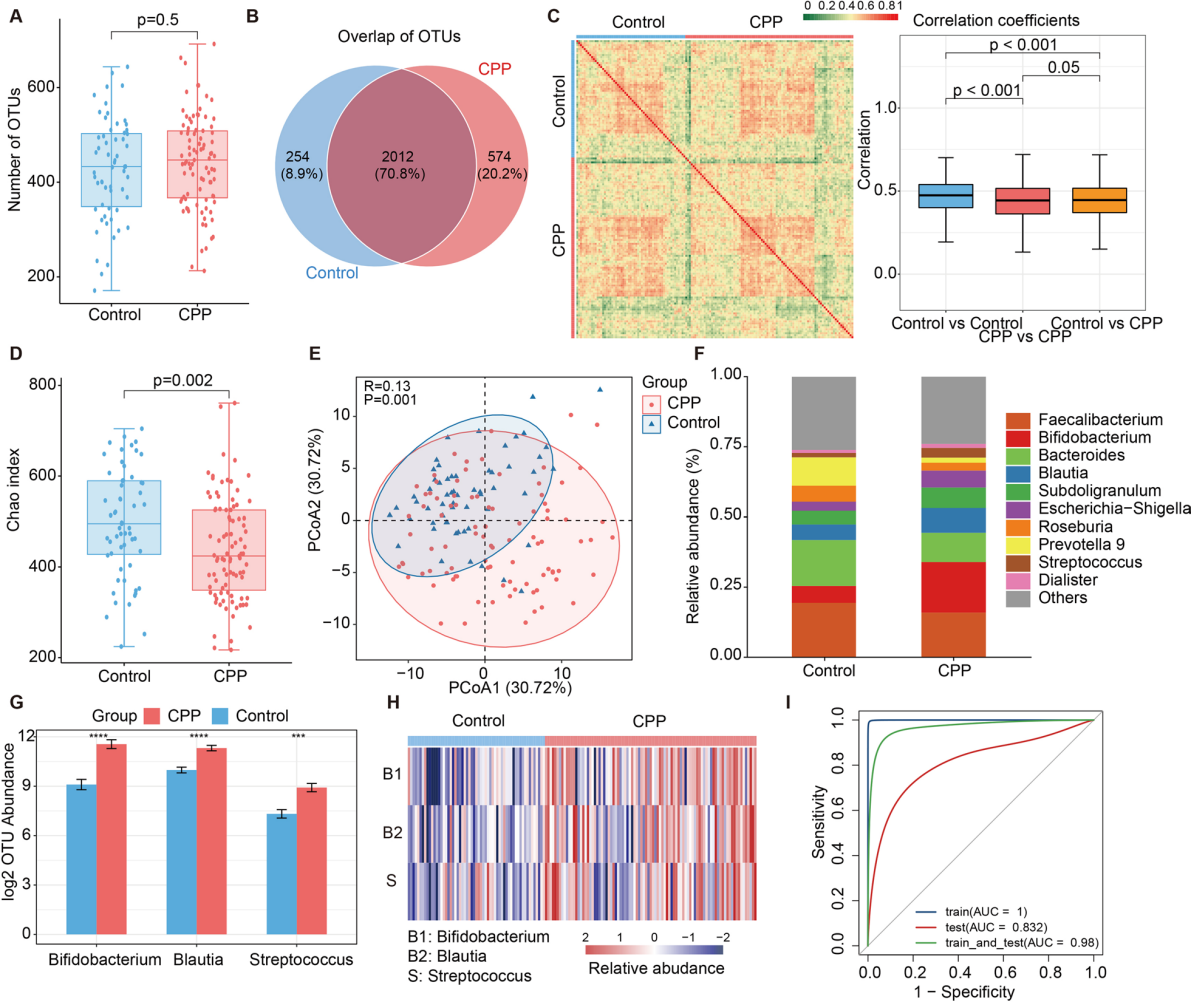
Gut microbial dysbiosis in CPP patients. **A** The number of OTUs between CPP group and healthy control group. **B** The shared OTUs between CPP group (shown in red) and healthy controls (shown in blue) (CPP group with 2586 and healthy controls with 2266). **C** The correlation analysis between two groups at the OUTs level. The heatmap on left showed the Spearman correlation coefficients and the box plots showed the correlation coefficients within and across group comparisons. **D** The distribution of the Chao index between two groups, and the healthy controls presented a higher Chao index (using the Wilcox’s rank sum test to calculate *p*-values). **E** PCoA analysis was performed based on the Bray–Curtis distance between two groups and the significant differences between groups were calculated by ANOSIM. This figure presented the first principal component and the second principal component. **F** The distribution of Genus species in two groups. **G** The differential analysis of species between two groups at the genus level. **H** The differences in species abundance between groups at the genus level. **I** The performance assessment of the random forest model based on the training set, test set, and training–testing set (****p <  = 1.0E^−4^, ***p <  = 0.001, **p <  = 0.01, *p < 0.05)

To investigate whether the alterations in gut microbiome are correlated with CPP, we performed 16s rRNA gene sequencing for a Chinese cohort containing 91 patients and 59 healthy controls. More than 10,000,000 sequences were annotated into the SILVA rRNA library database and subsequent analysis was carried out in the operational taxonomic unit (OTU). In this cohort, a total of 2840 OTUs were identified under a 97% similarity threshold condition. The number of OTUs in the CPP and healthy control groups was comparable that possibly due to a large number of shared microorganisms between the two groups (Fig. 1A–B). Despite the presence of considerable shared microorganisms, the OTU correlation within-group was higher than across groups **(**Fig. 1C**)**. The Chao index, as one of the indicators of alpha diversity, showed a lower level in the CPP group (Fig. 1D, p = 0.0021). At the same time, the analyses of the beta diversity using ANOSIM in combination with principle coordinated analysis (PCoA) also found significant differences across groups **(**Fig. 1E**)**. These results indicated that the composition of gut microbiota may have changed during CPP development.

Therefore, we compared the abundance of gut microbiota between CPP and healthy control groups. We found that Bifidobacterium, Blautia, and Streptococcus showed higher abundances in the CPP group at the Genus level (Fig. 1F–H). Since the differential species of gut microbiota identified between groups could not well distinguish CPP patients from the population, we used the random forest model and boruta algorithm to construct classifiers for all species. Finally, 24 representative species were identified and the performance assessment of the training set, test set, and training–testing set presented high classification efficiency, the AUC was 1.00, 0.932, and 0.98, respectively **(**Fig. 1I**)**. Overall, we identified different species between CPP and healthy control groups and constructed classifiers using random forests for distinguishing CPP patients.

### Functional pathways altered by gut microbiota in CPP patients

**Fig. 2:**
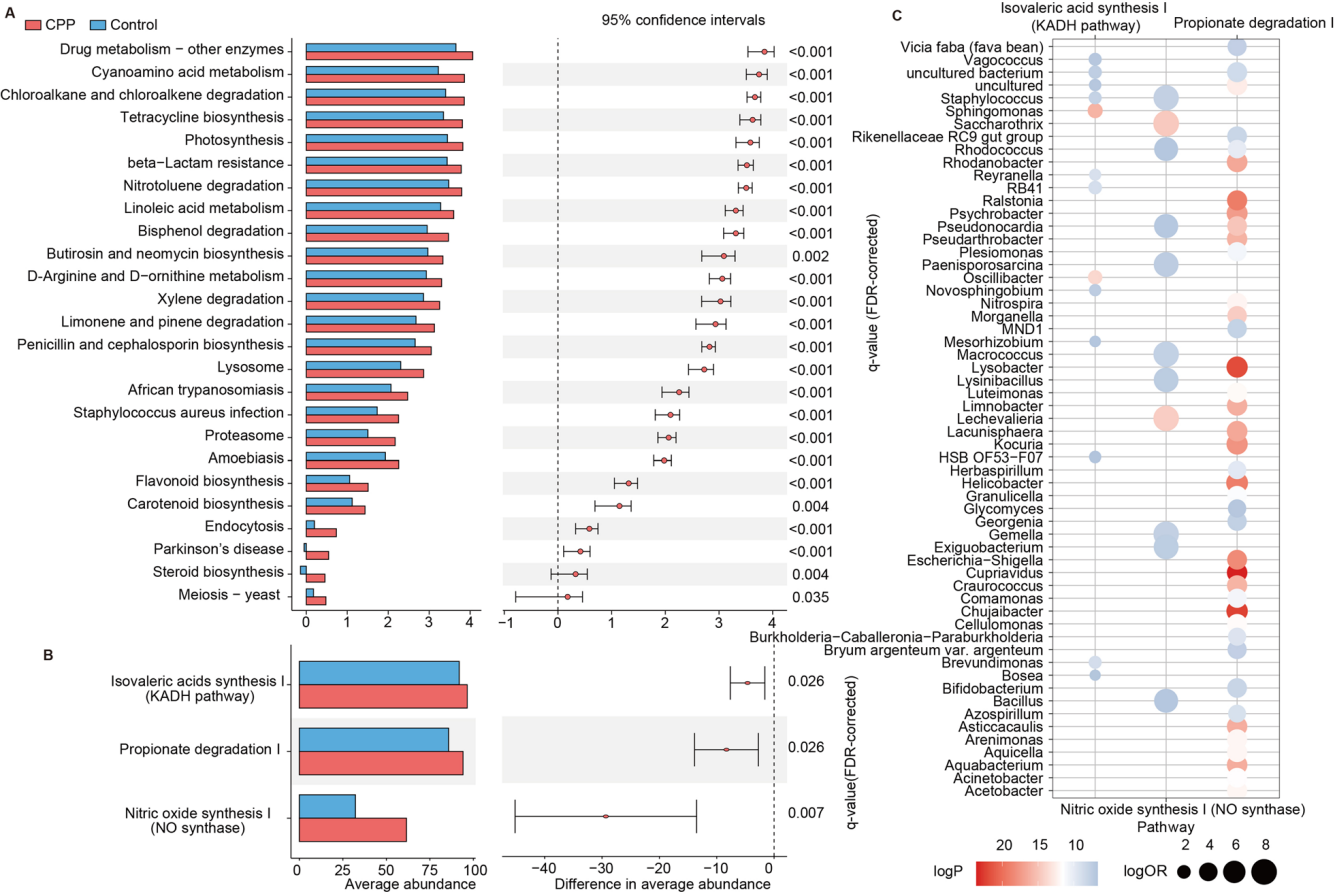
The functional analysis of gut microbes. **A** The average abundance of KEGG pathways with significant differences. *T*-statistics and *p*-values of differences in pathway activities between groups were calculated with *t*-test. **B** The average abundance of significantly different GBMs between two groups and *t*-statistics and significance (*p*-values) of *t*-test were presented. **C** The contributions of species to pathways at the genus level. *P*-values representing significance and odd ratios (OR value) were calculated using Fisher’s exact test

Next, we employed PICRUSt analysis to predict metabolic pathway activity and gut-brain module (GBMs) activity associated with neuroendocrine [20, 22]. Of the 173 metabolic pathways, we found that there were 25 pathways existed with significantly higher activities in CPP **(**Fig. 2A**)**, including Tetracycline biosynthesis, Bisphenol degradation, Lysosome, and Flavonoid biosynthesis. It was reported that the detection rate of antibiotics in the precocious puberty group was significantly higher than in adolescent children [23], and antibiotic exposure could result in disorders of gut microbiota [24]. As the one of the antibiotics, and the perturbations of tetracycline’s synthetic pathway may be related to the disorders of gut microbiota. As an endocrine disruptor, bisphenol has been demonstrated in previous studies to be associated with idiopathic CPP in 6-year-old girls [25]. Metachromatic leukodystrophy (MLD) caused by lysosomal abnormalities, which resulted from decreased activity of the enzyme arylsulfatase A and accumulation of aliphatic glucosinolates in the nervous system. The abnormal accumulation may interfere with the complex network that regulates the hypothalamic-pituitary axis, thereby inducing CPP [26]. The flavonoids, such as isoflavones whose biosynthesis could affect the kisspeptin signaling pathway, which may become the basis for precocious puberty in females [27].

Similarly, we also found that some GBM-related pathways exhibited higher activity in the CPP group **(**Fig. 2B**)**, for example, isovaleric acid synthesis, propionic acid degradation, and the synthesis of nitric oxide (NO). Notably, NO had an important effect on gaseous neurotransmitter that stimulates the secretion of gonadotropin-releasing hormone [28]. Afterward, we researched the contribution of species to GBMs through OUT based on the Genus level. The NO module was mainly contributed by 11 species, including Bacillus, Paenisporosarcina, and Rhodococcus **(**Fig. 2C**)**. These results suggested that alterations in the gut microbiota could influence the specific functions that have an impact on early puberty.

### Fecal metabolites alterations in CPP patients

**Fig. 3:**
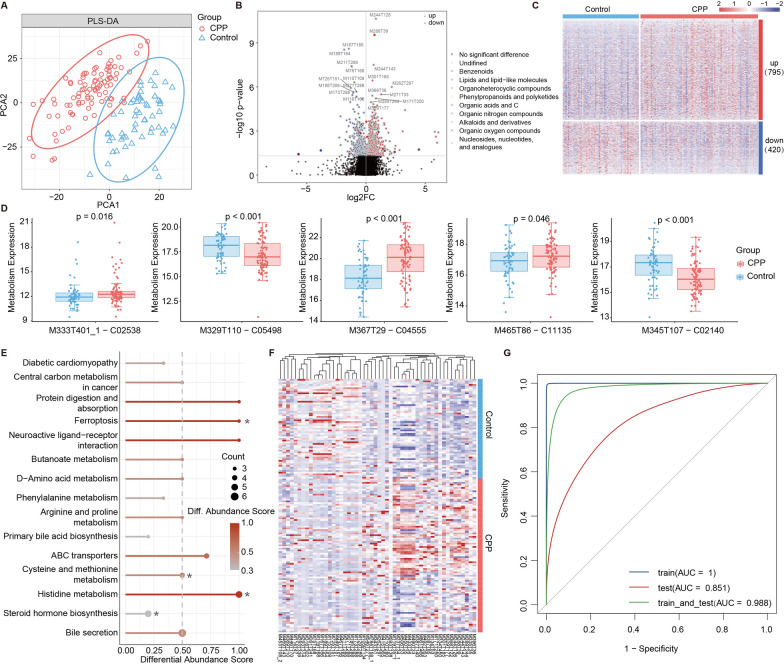
The altered fecal metabolites in the positive ion mode of the CPP group. **A** PLS-DA of CPP and healthy control groups. **B** The differential analysis between groups identified 1215 metabolites with significant differences and up-regulated and down-regulated metabolites with the top 20 largest differential degrees were marked. The outer circle colors of the dots indicated the different classifications of metabolites. **C** The abundance of 1215 metabolites. **D** The comparison of metabolites in steroid metabolic pathways between CPP and healthy controls. **E** The enrichment analysis of KEGG metabolic pathways based on differential metabolites and the enrichment significances were computed by hypergeometric distribution. The size of the point represented the enriched metabolites in pathways and the color of the point represented the differential abundance scores (*p < 0.05). **F** The abundance of the characteristic metabolites under positive ion mode of the classifier. **G** The performance evaluation of the random forest model according to the training set, test set, and training–testing set

To further refine the characteristics of CPP, we carried out nontargeted metabolomics profiling of fecal samples from 150 individuals (including 91 CPP patients and 59 healthy controls) and identified 15,411 compounds likely from the microbiome and host (including 8949 positive ions and 6771 negative ions). The partial least-squares discriminant analysis (PLS-DA) revealed a significant separation of positive and negative fecal metabolites between CPP and healthy control groups (Fig. 3A and Additional file 1: Fig. S1A). Comparing the abundances of positive ionic metabolites between CPP and healthy controls, we discovered 1215 metabolites were expressed differently in CPP, of which 795 were up-regulated and 420 were down-regulated. These metabolites covered lipid-like molecules, organic nitrogen compounds, phenyl-propanol, and polyketide (Fig. 3B–C). In addition, there were 1255 negative ion metabolites that differed significantly were identified via the same method, which consisted of 695 up-regulated and 560 down-regulated (Additional file 1: Fig. S1B–C).

To further understand the functions of these differential metabolites and their effects on the host, we next performed functional enrichment analysis and differential abundance (DA) scores [29] to capture the up or downregulation trend of pathway metabolites compared with healthy controls. Finally, fifteen pathways of at least 3 metabolites were identified in our analysis, of which 10 showed upward trends (Fig. 3E, DA >  = 0.5). These pathways over-activated in CPP were mainly involved in cysteine and methionine metabolism, histidine metabolism, and neuroactive ligand-receptor interactions **(**Fig. 3E**)**. Although steroid metabolism did not exhibit the same trend **(**Fig. 3D**)**, three metabolites were highly expressed in CPP, they were perturbed in CPP **(**Fig. 3E**)** and may affect the treatment of precocious puberty [30]. These results indicated that metabolite alterations and functions are related to CPP.

Therefore, we used positive ion metabolites to construct a classifier and recognized 52 characteristic metabolites **(**Fig. 3F**)**. The performance evaluation of the training set, test set, and training–testing set based on the positive ion classifier presented better classification efficiency, whose AUC was 1, 0.85, and 0.988, respectively **(**Fig. 3G**)**. In addition, the performances based on the negative ion classifier were all high (Additional file 1: Fig. S1D). The good performance of the RF classifiers constructed separately for positive and negative ions indicates that either one can be used to classify the CPP samples. Overall, we identified differential metabolites between CPP and healthy control groups and constructed a random forest classifier based on the fecal metabolite.

### Blood metabolomics alterations in CPP patients

**Fig. 4:**
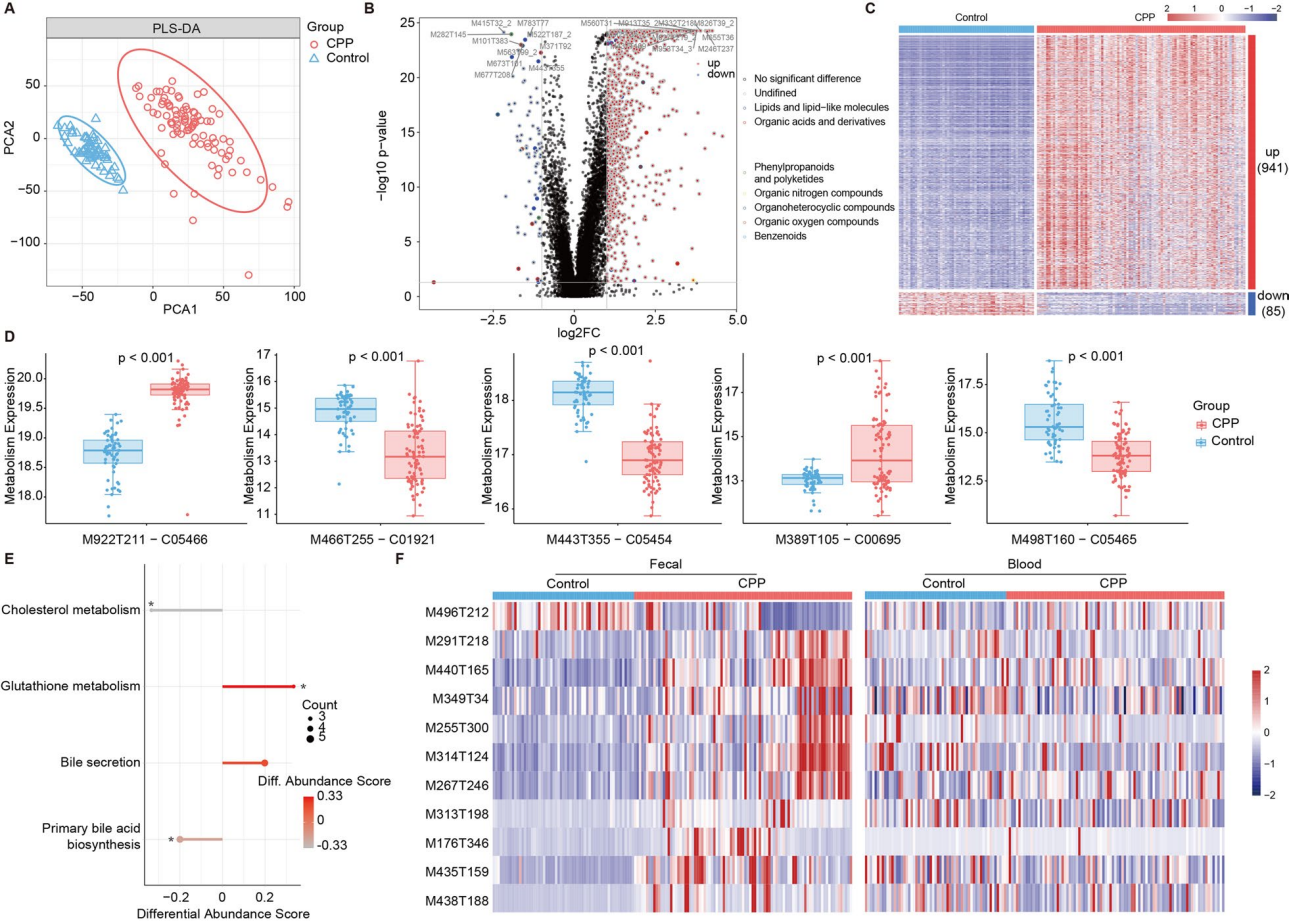
The altered blood metabolites in the positive ion mode of the CPP group. **A** PLS-DA of CPP group and healthy controls. **B** The analysis of differences between groups recognized 1026 metabolites with significant differences and up-regulated and down-regulated metabolites with the top 20 largest differential degrees were marked. The outer circle colors of the dots indicated the different classifications of metabolites. **C** The abundance of 1026 metabolites. **D** The comparison of metabolites in primary bile acid biosynthesis pathways between two groups. **E** The enrichment analysis of KEGG metabolic pathways according to differential metabolites and the enrichment significances were computed by hypergeometric distribution. The size of the point represented the enriched metabolites in pathways and the color of the point represented the differential abundance scores (* *p* < 0.05). **F** The abundance of the shared metabolites in the fecal and blood

Similarly, we performed untargeted metabolomic analysis on blood samples from 150 individuals and recognized 18,867compounds (including 12,898 positive ions and 6193 negative ions) that may be from the microbiome and host. PLS-DA revealed positive and negative fecal metabolites were clearly separated between CPP and healthy controls (Fig. 4A, Additional file 1: Fig. S2A). We compared the abundance of positive ion metabolites between the two groups and discovered that 1026 metabolites were expressed differently in CPP of which 941 were up-regulated and 85 were down-regulated. These metabolites covered the classification of lipid-like molecules, organic nitrogen compounds, and benzene ring compounds **(**Fig. 4B–C**)**. Furthermore, 167 negative ions, including 108 up-regulated and 59 down-regulated, were recognized using the same method (Additional file 1: Fig. S2B–C). We next used positive or negative ion metabolites to construct the classifiers. We found that the RF classifiers constructed separately for positive and negative ions gave both good performances (Additional file 1: Fig. S3).

After analyzing the functional enrichment and assessing the differential abundance of these metabolites, we captured four pathways of at least 3 metabolites of which two showed an increasing trend (DA >  = 0.2) and two presented a decreasing trend (DA <  = − 0.2). Interestingly, these pathways decreased in CPP were involved in cholesterol metabolism and primary bile acid biosynthesis (Fig. 4D–E**)**. However, cholesterol and bile acid were used to treat precocious puberty [31]. These results indicated that the changes and functions of blood metabolites were associated with CPP. Fecal and blood metabolites possessed different expression abundances in the CPP group and healthy controls, and the expression of these metabolites appeared to be more tendentious in fecal samples (Fig. 4F).

### Associations of gut bacterial and fecal metabolites in CPP

**Fig. 5:**
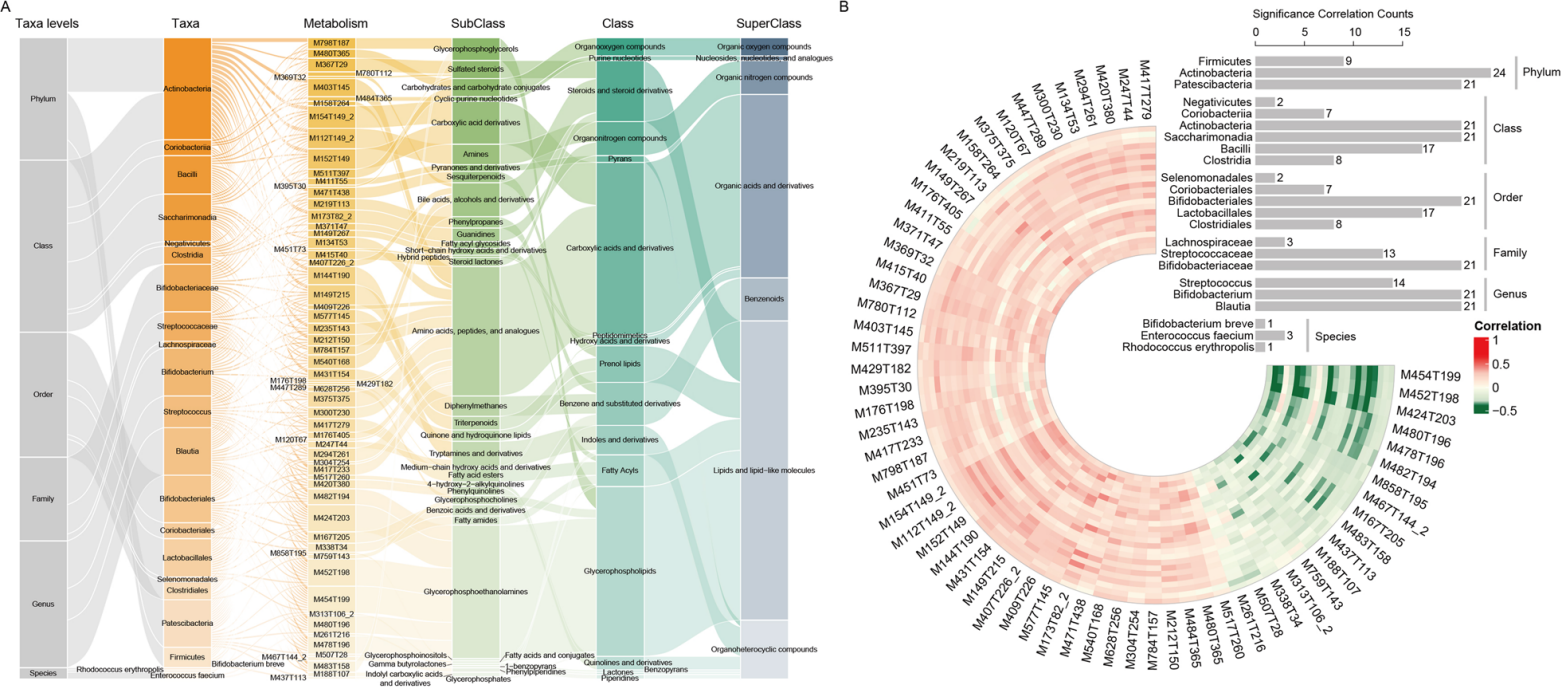
The fecal metabolic potential of gut microbial in CPP group. **A** The intestinal microorganisms-metabolites interactions and the categorization of metabolites. The color shades indicated the differential extent of microorganisms or metabolites between groups. **B** The circus plot displayed the correlation coefficients between 23 gut microorganisms and 64 metabolites, and the bar graph showed the number of metabolites significantly related to the microbe at 6 levels

To obtain the metabolic potential of gut microbiota in the CPP group, we calculated Spearman correlation coefficient of differentially expressed microorganisms and fecal metabolites in CPP group at 6 taxonomic levels (including Phylum, Class, Order, Family, Genus and Species), respectively. This procedure utilized metabolites with annotated names after positive and negative ions modes were merged. Among the 262 differential metabolites, we observed that 64 metabolites were with strong correlations with 23 gut microbes (Spearman correlation coefficient >  = 0.3 or <  = − 0.3, BH corrected *p*-values < 0.05). These metabolites were involved in lipids and lipid-like molecules, organoheterocyclic compounds, Benzenoids, organic acids and derivatives, organic nitrogen compounds, organic oxygen compounds, Nucleosides, nucleotides, and analogues (Fig. 5A). Specifically, the genus species Streptococcus and Bifidobacterium played promoted role in sexual development [32] and upregulated in CPP. Bifidobacterium was found to be correlated with 21 metabolites (Fig. 5B), including M149T215 (Bisphenol b) and M431T154 (alpha-Tocopherol (Vitamin E)), which showed positive correlations. Bisphenol b has been shown to disrupt the uterine immune landscape in mice. Furthermore, it has been reported that Vitamin E plays an important role in hypothalamic control of luteinizing hormone-releasing hormone (LHRH) and ascorbic acid (AA) release in mice by mediating the release of NO [33].

### Associations of gut bacterial and blood metabolites in CPP

**Fig. 6:**
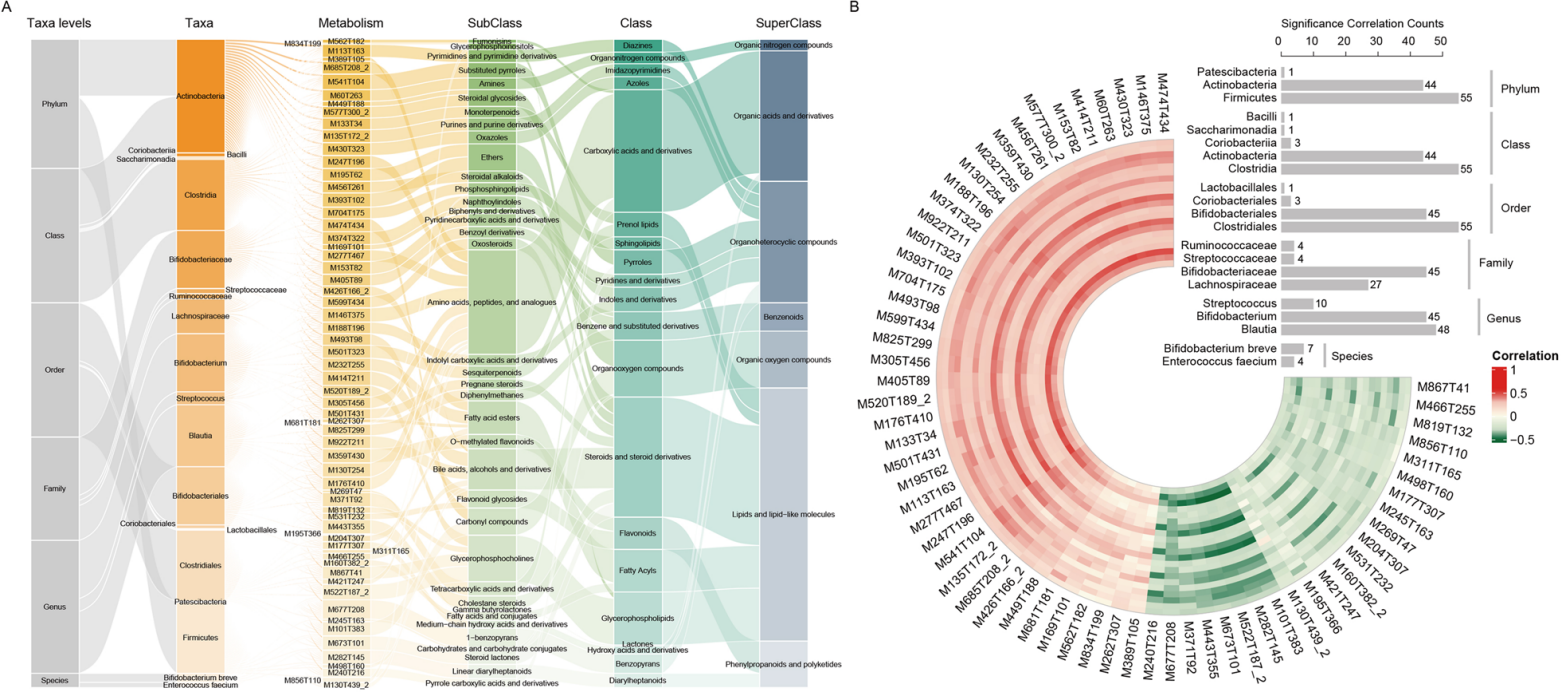
The blood metabolic potential of gut microbial in CPP group. **A** The interactions between gut microorganisms and metabolites, and the classification of metabolites. The color shades represented the differential extent of microorganisms or metabolites differences between groups. **B** The circus plot showed the correlation coefficients between 21 gut microorganisms and 64 metabolites. The bar graph exhibited the metabolites number significantly associated with the microbe at 6 levels

Likewise, we calculated Spearman correlation coefficient of differentially expressed microorganisms and differentially expressed blood metabolites at 6 levels in CPP group, respectively. This analysis also used metabolites with annotated names that were considering both positive and negative ions modes. Out of 99 differential metabolites, we observed that 64 metabolites had strong correlations with 21 gut microorganisms at 6 levels (correlation coefficient >  = 0.3 or <  = − 0.3, BH-corrected p-value < 0.05). These metabolites involved lipids and lipid-like molecules, organic acids and derivatives, phenylpropanoids and polyketides, and organic oxygen compounds, among others **(**Fig. 6A**)**. Consistent with the previous observations, we also found strong associations of the genus Blautia, Streptococcus, and Bifidobacterium, which exhibited higher abundances in CPP group, with M430T323 (Tubacin). Tubacin has been shown to significantly increase the expression of endothelial nitric oxide synthase [34], and NO is known to stimulate the secretion of gonadotropin-releasing hormone [28].

Furthermore, Blautia and Bifidobacterium were positively correlated with M247T196 (Tryptophan betaine, Fig. 6B). Tryptophan betaine has been identified as a strong predictor of vitamin D [35]. It has been suggested that vitamin D status may be associated with CPP risk and may have a threshold effect on CPP [36]. These results suggested that the dynamic changes of gut microbiota and the increase of Blautia, Streptococcus, and Bifidobacterium in the CPP group may influence NO-related fecal or blood metabolites, leading to CPP by stimulating the secretion of gonadotropin-releasing hormone. Overall, the alterations of metabolites associated with microbiota dysbiosis provided new insights for the diagnosis and treatment of CPP.

## Discussion

In this study, we have discovered the alterations in the characteristics of gut microbiota, fecal and blood metabolites in patients with CPP, and identified some microbial and metabolite candidates that may be useful for CPP treatment. Genus Bifidobacterium and Streptococcus were highly enriched in CPP, both of which were associated with signal transduction of GnRH [32, 37]. Fecal metabolites M333T401_1 (Estrone sulfate), M367T29 (3-dehydroepiandrosterone sulfate), M465T86 (Androsterone glucuronide), M329T110 (11beta-hydroxyprogesterone) and M345T107 (Corticosterone) showed a significant difference between CPP and healthy controls, and these five metabolites with a weak overall up-regulated tendency mediated steroid metabolism. Among of them, Estrone sulfate and Androsterone glucuronide were highly enriched in CPP, which correlated with GnRH activation [38, 39]. While Corticosterone was highly enriched in healthy controls, possibly due to its role in downregulating follicle-stimulating hormone and luteinizing hormone [40]. The blood metabolites M450T211 (Glycochenodeoxycholate), M466T255 (Glycocholate), M443T355 (Trihydroxycoprostane), M426T226 (Cholic acid), and M498T160 (Taurochenodeoxycholic acid) showed significant differences in CPP and healthy controls. These five metabolites with a generally downregulated trend mediated the primary bile acid biosynthesis pathway, among which the organic acid Glycocholate could promote the absorption of sex hormones [41]. The up-regulation of Glycocholate in healthy control group meant that Glycocholate in CPP patients may be degraded because of promoting the release of sex hormones.

In addition to a brief description of changes in gut microbes, fecal, and blood metabolites in CPP, our study also identified CPP-related KEGG metabolic pathways and neuroendocrine GBMs. Previous studies have reported that Tetracycline biosynthesis, Bisphenol degradation, Lysosome, Flavonoid biosynthesis, and NO synthesis pathway were associated with the treatment and pathogenesis of CPP [24–28]. During the functional annotations of fecal and blood metabolites, we discovered the dysfunctional pathways in disease progression and quantified the roles of metabolites in pathways using differential abundance scores. Such as cholesterol metabolism and primary bile acid biosynthesis enriched in blood metabolites, which were mediated by upregulated blood metabolites, while cholesterol and bile acids were discovered to be contributed to the treatment of precocious puberty [31].

Conventional studies on the pathogenesis of CPP have focused on host genetics and peripheral factors [42], and several studies have analyzed the gut microbiota in CPP [16], but only identified dysregulated gut microbiota and did not analyze downstream pathways. This study not only revealed dysregulated microbiota and metabolites in CPP through analysis of microbiome and metabolome, but also linked them together, aiming to characterize the influence of microbiota on the body using metabolites, providing a new perspective for the diagnosis and treatment of CPP. Together, the study of CPP requires the integration of multiple omics data to comprehensively depict the dynamic changes of response factors in the disease process and discover the pathogenesis.

However, we also realized several limitations of this study. Although 16 s rRNA sequencing was widely used to characterize microbial communities, it existed limitations in explaining complete genetic information compared to metagenomic sequencing. Nevertheless, 16 s rRNA gene sequencing was mature technology and was enough for massive research. Furthermore, for the studies of metabolites, candidate microorganisms need to be further cultured to judge the origin of metabolites more accurately. Precocious puberty was often related to obesity [43], but the population collected in this study did not contain people with obesity which made it impossible to explore the co-occurrence effect of microorganisms, metabolites, and obesity on CPP, but this direction deserves to be studied.

## Conclusions

In conclusion, we integrated for the first time microbiomics and metabolomics to characterize systematic changes in gut microbes, fecal and blood metabolites in CPP patients. We revealed the microbial and metabolite features associated with CPP, interpreted the correlation between the two in the setting of CPP, and developed a predictive model to distinguish and diagnose for CPP.

## Supplementary Information

**Additional file 1**: **Fig S1**. The altered fecal metabolites in the negative ion mode of the CPP group. **Fig S2**. The altered blood metabolites in the negative ion mode of the CPP group. **Fig S3**. The random forest models for blood metabolites in CPP group.

## Data Availability

The data obtained in the analysis of this article are included in this paper, and the raw data reported here are available upon requested to the corresponding authors.

## Abbreviations

CPP: Central precocious puberty
HPGA: Hypothalamic-pituitary–gonadal axis
GnRH: Gonadotropin-releasing hormone
HILIC: Hydrophilic interaction chromatography
IDA: Information-dependent acquisition
OTU: Operational taxonomic unit
GBM: Gut-brain module
NO: Nitric oxide
LHRH: Luteinizing hormone-releasing hormone
