# Supplementary material for: Gut microbiome combined with metabolomics reveals biomarkers and pathways in central precocious puberty
Source: J Transl Med. 2023 May 11;21:316. doi: 10.1186/s12967-023-04169-5 (PMC10176710; doi:10.1186/s12967-023-04169-5)
Supplement: Supplementary file 1 — Additional file 1: Fig S1. The altered fecal metabolites in the negative ion mode of the CPP group. Fig S2. The altered blood metabolites in the negative ion mode of the CPP group. Fig S3. The random forest models for blood metabolites in CPP group. [file 12967_2023_4169_MOESM1_ESM.docx]

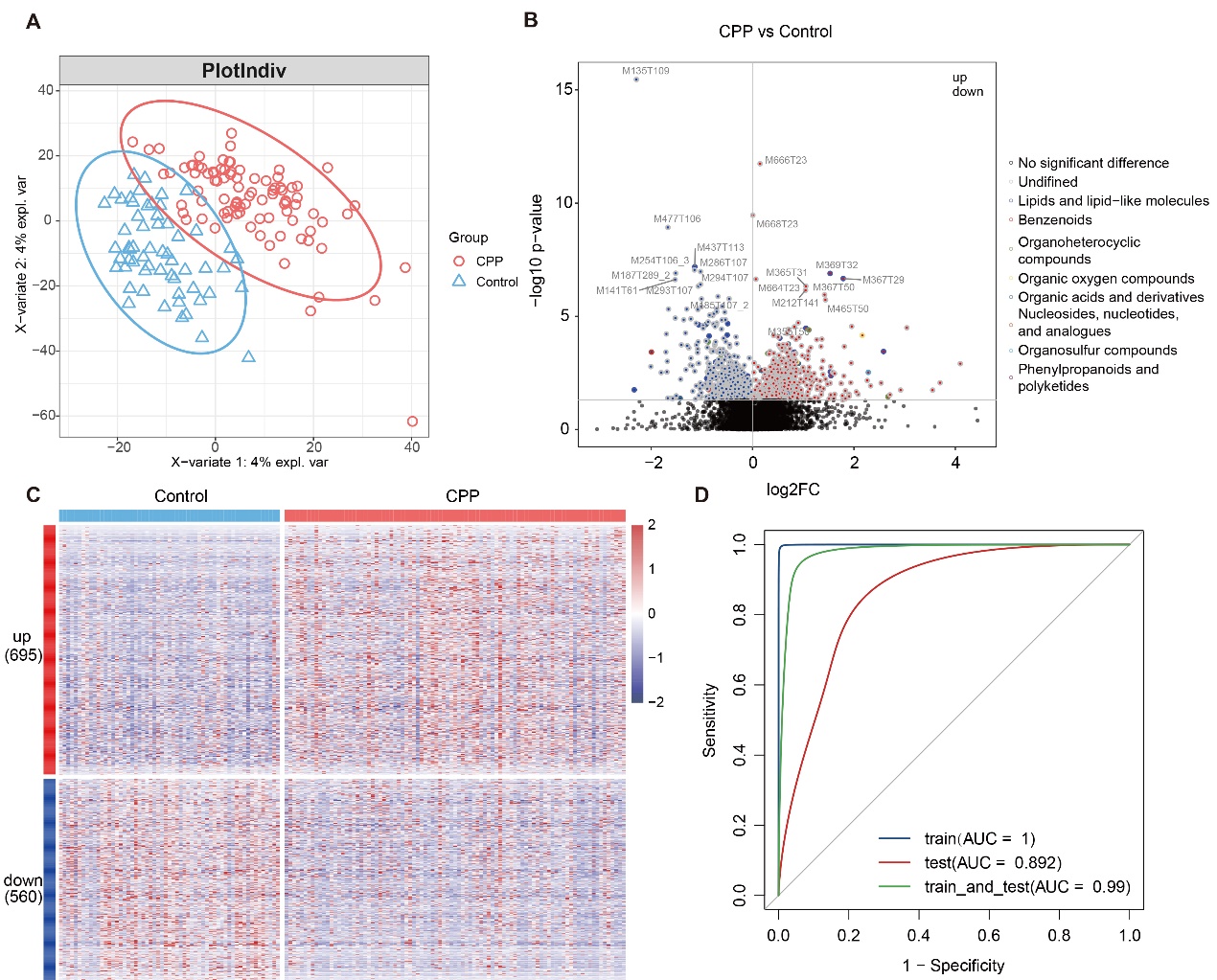


**Fig. S1. The altered fecal metabolites in the negative ion mode of the CPP group.** (A) PLS-DA of CPP and healthy control groups. (B) The differential analysis between groups identified 1255 metabolites with significant differences and up-regulated and down-regulated metabolites with the top 20 largest differential degrees were marked. The outer circle colors of the dots indicated the different classifications of metabolites. (C) The abundance of 1255 metabolites. (D) The performance evaluation of the random forest model according to the training set, test set, and training-testing set.


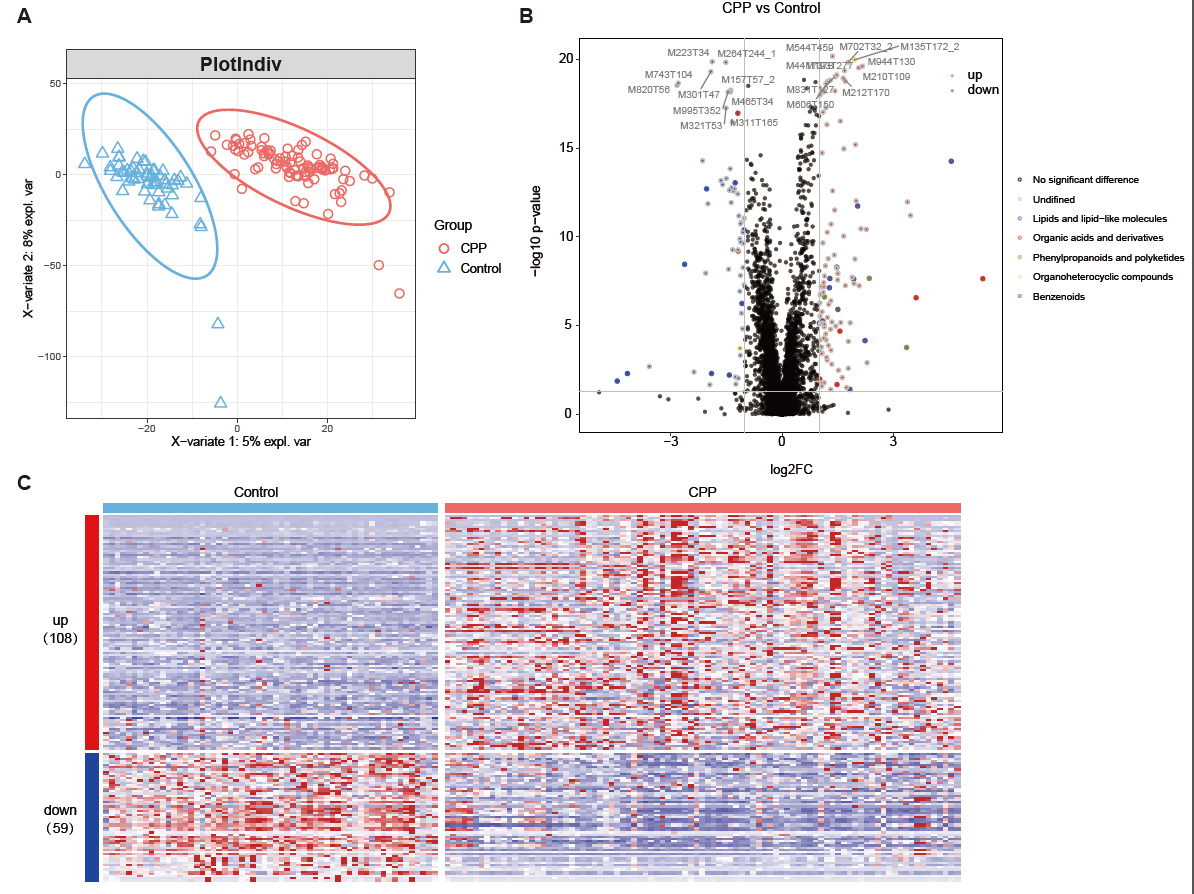


**Fig. S2. The altered blood metabolites in the negative ion mode of the CPP group.** (A) PLS-DA of CPP and healthy control groups. (B) The differential analysis between groups identified 167 metabolites with significant differences and up-regulated and down-regulated metabolites with the top 20 largest differential degrees were marked. The outer circle colors of the dots indicated the different classifications of metabolites. (C) The abundance of 167 metabolites.


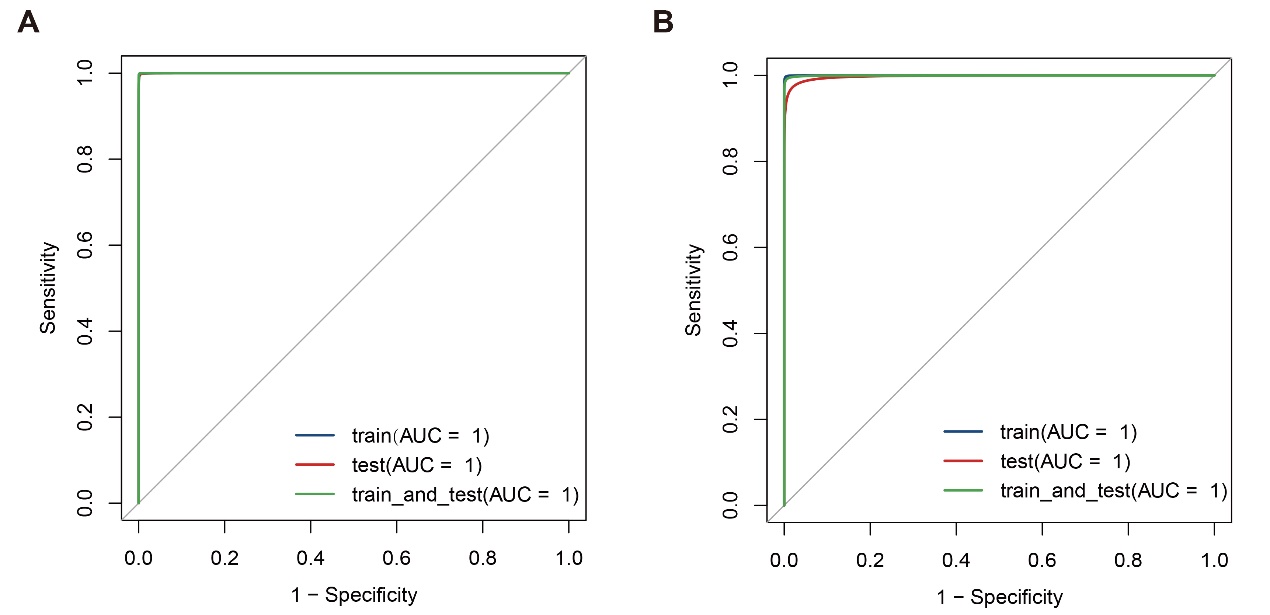


**Fig. S3. The random forest models for blood metabolites in CPP group.** (A) The performance evaluation of the random forest model for positive metabolites according to the training set, test set, and training-testing set. (B) The performance evaluation of the random forest model for negative metabolites according to the training set, test set, and training-testing set.
